# Medical Items and News of Interest to the Physician

**Published:** 1884-04

**Authors:** 


### For the Use of Physicians.

CULLED FROM THE STANDARD MEDICAL JOURNALS OF THE WORLD.

NOTE.—These formulæ are intended only for the regular practitioner of medicine, and should be employed only by him, or under his immediate supervision.

**Oxide of Zinc as a Substitute for Iodoform.**

In the treatment of wounds, Dr. Peterson, of Kiel, considers zinc oxide a good substitute for iodoform. It is cheaper, and is not poisonous.

**Scabies.**

Guerin applies an ointment made by dissolving ten parts of naphthol in five parts ether, and mixing it with the one hundred parts of vaseline. Secluded from the air it will keep a long time.

**Colic.**

Dr. A. Tepliashin recommends a thin stream of cold water from a teapot lifted from one to one and a half feet from the abdomen, in cases of colic. He has seen it relieve pain when opium and morphia had failed.

**Croup.**

Dr. Meunier reports favorably a number of cases of croup treated by sulphide of calcium, in doses of from three to four and one-half grains daily. The granules, one-tenth grain each, are used one or two every hour.

**Ephelis.**

Take of sulpho-carbolate of zinc one part, of collodion forty-five parts, of oil of lemon one part, of absolute alcohol five parts. The sulpho-carbolate should be reduced to a fine powder and thoroughly admixed.—*Chicago Med. Review.*

**Typhoid Fever.**

Dr. Dicplal, says the *American Journal of Pharmacy*, after long experimentation with various salicylates in typhoid fever, has found the salicylate of bismuth the great desideratum. In his experience it has even had a marked abortive action. Out of twenty cases reported by him, eleven treated in the first stage were able to be about in four or five days under the free use of salicylate of bismuth. The ordinary dose is about a scruple. This was repeated, so that the daily quantity taken should equal about six grammes.

**Vesical Catarrh.**

Dr. Boegehold is inclined to believe that salicylic acid is as much of a specific for rheumatic and gonorrhœal catarrh as it is for articular rheumatism. When chlorate of potash has been of no avail, he has found salicylic acid and salicylate of soda to effect a cure.

**Neuralgia.**

Dr. Rasori recommends the use of the tuning-fork in the treatment of neuralgic pains, the instrument to be applied, while vibrating, over the course of the painful nerve. The sittings are said to last about half an hour, when the pain is usually relieved without further treatment.

**Schuyler's Powder.**

This is a sort of Dover's powder, composed as follows:

Sulph. morphine, 1 part.
Camphor, 6 parts.
Ipecac, pow'd, 6 parts.
Glycyrrhiza, pow'd, 25 parts.
Sugar, pow'd, 25 parts.

—*Rev. farm. and Ph. Post.*

**Perosmic Acid.**

This is a new remedy employed by Professor Winiwarter in cancerous and scrofulous swellings. It is used by injecting daily three drops of a one per cent. solution of the acid, which treatment causes the tumor to soften and decrease in size, the dead tissue is thrown off, and disappears in about a month. No curative effects upon cancer itself have been observed from the remedy.

**Colds—Hot Water in the Treatment of.**

Dr. George R. Sheppard, Hartford, Conn., says, in respect to the use of hot water as a remedial agent in the treatment of inflammation of the mucous membranes: "I have used hot water as a gargle for the past six or eight years. In acute pharyngitis and tonsillitis, and in coryza, or cold in the head, if properly used in the commencement of the attack, it constitutes one of our most effective remedies, being frequently promptly curative. To be of service it should be used in considerable quantity (a half pint or a pint at a time), and just as hot as the throat will tolerate. I have seen many cases of acute disease thus aborted, and can commend the method with great confidence."

**Winter Eczema—Squibb's Formula.**

For the relief of winter eczema, a troublesome itching affection, Dr. Squibb recommends the following: Take of tannic acid, forty grains; of glycerine and alcohol each half an ounce; water to make four ounces. This solution is applied to the itching surfaces by means of a small sponge or rag, morning and evening.—*Detroit Lancet.*

---

**Quinine.**

Professors Bartholow and Da Costa agree that an antipyretic dose of quinine is not less than five grains every two hours, until four doses are taken, or else thirty grains in two or three doses close together. The former believes a small dose of morphine given with quinine is the best thing to counteract the unpleasant cerebal symptoms of the latter.—*Louisville Medical News.*

---

**Diphtheria.**

Dr. J. P. Lyttle, in *Chicago Medical Journal and Examiner*, says that the spray of dilute lactic acid will dissolve the pseudo-membrane of croup. He has used it in a number of cases with the happiest results. He sprays the throat with a solution of concentrated lactic acid, thirty drops to one drachm of water. Under this spray the membrane is rapidly dissolved and disappears.

---

**Cystitis.**

Dr. J. Andeer, *Centralbl. f. die Med. Wissen.*, reports extensive use of *resorcin* in acute and chronic cystitis, and claims for it almost specific curative power. He reports one hundred and fifty-six cases where, either by him or to his personal knowledge, it was injected into the human bladder with the best results in vesical catarrh. Acute cases have been entirely cured by the injection of a five per cent. solution.

---

**Skin Diseases.**

M. Desguin, of Antwerp, gives glycerine internally in certain forms of skin disease, with, it is said, marked success, especially in acne punctata and the furuncular diathesis. He commences with four drachms daily, and gradually increases the dose. He states that the secretion of the cutaneous glands, which is thick and irritating in these diseases, becomes more liquid, and cutaneous irritation is notably lessened. During convalescence from scarlet fever he believes that it facilitates desquamation.

**Puerperal Eclampsia.**

Dr. Breus highly recommends his plan of treatment of puerperal convulsions (*Med. and Surg. Reporter*), which consists in hot baths and wrapping in blankets until profuse diaphoresis occurs. He has never seen any evil results, and thinks it desirable to employ this treatment in pregnant women who are the subjects of dropsy or albuminuria, believing that by it the onset of eclampsia may possibly be prevented.—*Weekly Med. Rev.*

---

**Lupus.**

Dr. Duhring, of Philadelphia, recommends the following formula as valuable for a wash in lupus erythematosus:

℞

Zinci sulphatis,

Potass. sulphidi, aa ʒ ss.

Aquæ rosæ, ℥ iiiss.

Alcohol, ʒ iij.

With the occasional addition of a few minims of glycerine.—*Phil. Med. Times.*

---

**Tannate of Sodium.**

Tannate of sodium has been lately recommended instead of tannin to lessen the excretion of albumen in albuminuria; but the statements concerning its value are somewhat conflicting. Ribbet found that it lessened the excretion of albumen in animals. Dr. Brien, on the other hand, found, in four carefully observed cases of patients suffering from chronic albuminuria, that it was of no use whatever; some patients can take it well; others vomit after every dose.—*Practit.*

---

**Oleate of Quinine.**

Acting on the suggestions of Dr. John V. Shoemaker in relation to oleic acid and its combinations, Dr. Thomas F. Wood has tried the efficacy of the oleate of quinine, a report of the cases being published in the *North Carolina Medical Journal.* This remedy was used in two cases, both being children suffering from gastro-intestinal derangements, and cinchonism followed its application. In these cases the drug was applied locally, at first to the inside of the thighs and arm-pits, afterwards all over the abdomen, and in this way in the course of twenty-four hours 60 grains of alkaloid were used. Clinical observation showed a reduction of the temperature, but the results are not to be considered as positively due to the quinine.

**Ulcers.**

Dr. Maison regards subcarbonate of iron as the best remedy for the local treatment of ulcers of various kinds, even those of syphilitic origin. The mode of application is as follows: The surface of the ulcer is first washed with carbolized water and then dusted thickly with powdered subcarbonate of iron, and over this is put a starch poultice. The dressing is usually changed twice a day. The healing process is very rapid, and has even taken place in ulcers which were rebellious to iodoform.

---

**Epistaxis, Persistent.**

There are few things more trying to the young doctor (and to the old one too) than a persistent hemorrhage of the nose. As the blood keeps dropping, dropping, dropping, so equally do the physician's spirits. Dr. Guedevo tells us in *L'Indipendente* that he succeeded in controlling two cases with the following, given in teaspoonful doses every fifteen minutes. After some vomiting the hemorrhage ceased: Tartrate of antimony and potash, 2 grains; distilled water, 4 fluid ounces; simple syrup, 6 fluid ounces.

---

**Cancer Remedy.**

A writer in the *Br. Medical Journal* speaks highly of "cleavers" (*Galium Aparine*) as a cancer remedy. He claims that a local application reduces the size and relieves the pain of the cancer, and writes that in Hertfordshire it is used also internally. The mode is to give an aperient and enjoin a simple diet; the patient to tak ℥ v. of the juice of the plant twice daily. At the same time an ointment of the juice should be applied to the sore, frequently renewed. Though recovery is gradual, it is claimed that one ulcer was healed in three months.

---

**Sugar as a Dressing for Wounds.**

Professor Lucke, at Strasburg, and Dr. Windelschmidt, at Cologne, report favorably on the use of powdered cane sugar as an antiseptic dressing for wounds. It has been used in connection with naphthalin (equal parts) or with iodoform (1 part, sugar 5 parts), or alone. The only difficulty so far, is found to be the tendency of the patients to mistrust the dressing when they discover its nature, and on the other hand, to treat themselves, and pass from under observation.

NOTE.—Was it not the iodoform or naphthalin that did the work?—ED.

**Dilatation of the Heart.**

Professor Maraglianô formulates the results of the exhibition of strychnine in cardiac dilation as follows:

First—In one or two days the size of the heart was reduced, and in five or six days very considerable dilatations were caused to disappear.

Second—If, immediately upon a reduction in size of the heart, the strychnine were withheld, the dilatation was frequently reproduced.

Third—The daily dose of sulphate strychnine required was from $\frac{1}{32}$ to $\frac{1}{20}$ grain.—*Memorabilien.—Med. Record.*

---

**Mycotic Disease of the Kidneys.**

Dr. M. Litten, in the *Ztsche. f. Klin. Med.*, roports two very interesting cases of mycotic affection of the kidneys. Two persons living together in one house became suddenly affected with rigors, great increase of temperature and albuminuria of a high degree. Under uræmic symptoms both died. Toward the end perfect anæmia was present. At the *post-mortem* the kidneys were found filled with bacteria. Both patients had been drinking water from a well which existed near a grave-yard. The water was found full of bacteria. Similar cases have been reported this year by Bamberger and Aufrecht.—*Chicago Med. Rev.*

[Another eloquent argument for crematfon.—ED. BISTOURY.]

---

**Coffee—Its Physiological Action.**

The *Repertoire de Pharmacie* gives a description of the physiological action of coffee, our common beverage, by Guimaraes. His observations were made on dogs. Ordinary doses of coffee cause an increase in the heart action, increase the blood pressure, accelerate respiration, elevate the body temperature, and slightly increase the irritability of the nervous centers. To produce the opposite effects, it requires very large doses. This beverage can be recommended, first, because it increases the assimilative powers of the system; and second, as a stimulant it is superior to alcohol, since the after-effects are not so unpleasant. Even in large doses the balance between assimilation and elimination is always kept up. We are still in the dark concerning many points yet, but we know positively why coffee is useful to individuals who are compelled to labor for a long time, because it increases the ability for work by its stimulating properties.

**Dyspepsia.**

℞

Tinct. nux vomica, ♏ v.

Powd. ipecac, gr. ¼.

Bicarb. soda, grs. v.

Powd. rhubarb, grs. ij.

Peppermint water, q. s., ʒ j.

M. Dose, ʒ j.

PEPSINE.

℞

Saccharat. pepsine, grs. iij.
Dil. muriat. acid, ♏ iij.
Glycerine, ♏ v.
Water, s. q., ʒ j.

M. Dose, ʒ j.

---

**Eczema.**

In the eczema of the scalp in children, Dr. Lassar recommends, after cleaning the surface:

℞

Acid. salicylic, 1 g.
Tinct. benzoini, 2 g.
Ung. petrolei, 50 g.

M. To be employed two or three times a day.

In eczema of the non-hairy portions he employs:

℞

Acid. salicylic, 2 g.
Ung. petrolei, 50 g.
Zinci oxidi,
Amyli, aa 25 g.

M. This paste is absolutely unirritating, and, besides, has the advantage that it does not retain the exudation upon the skin, but allows it to escape.—*Centralblatt fur Chirurgie.*

---

**Constipation.**

It has been observed, as a result of the poisonous action of calabar bean on animals, that there is a tetanic spasm of the muscular coats of the intestines, which results in the forcible expulsion of the contents. This physiological property of the drug suggested to Dr. Schaefer (*Berlin. klin. Wochenschrift*) the employment of the drug in cases of obstinate constipation), dependent on weakness of the muscular coats of the intestines, such as may be frequently met with in women and old men. The results of his experiments have amply justified his anticipations, based on the physiological properties of the drug, severe cases having yielded to the treatment in less than twenty-four hours after its administration. His formula consists of a solution of ⅚ of a grain of extract of physostigma in 2½ drachms of glycerine. Of this six drops are given every three hours.

**Blood Diet.**

As a result of five years' experience at the Royal Hospital for children and women, Dr. Ed. O. Day gives, in the *Lancet*, the following mode of preparing blood as a diet for delicate children:

A given quantity of blood is collected, and before coagulation takes place, is mixed with three parts of water, and kept in constant movement for some time, then gradually evaporated over a water bath, at a temperature not exceeding 100° F. Very great care has to be taken to prevent coagulation. When the consistence of chocolate is reached, a higher temperature may be used to perfect drying. It should be reduced to a powder in a mortar. It is like coffee, with very little taste, but with a peculiar odor. If properly prepared it should be perfectly soluble in water. He commences, for a child one year old, with a teaspoonful during the day.—*Med. and Surg. Rep.*

---

**Coryza.**

The power possessed by atropine of diminishing the secretion of the nasal mucous membrane led Gentilhomme to employ it in acute coryza. In several severe cases, characterized by profuse secretions, fever, and difficulty of breathing, which in one case closely resembled asthma, one-half milligram of atropine given in pill form served to arrest the disease entirely. When the disease has passed through the acute stage, its administration improves the general condition, but its effect is not as striking as when given in the acute stage. Lubliski also reports decided improvement resulting from the administration of one milligram of the sulphate of atropine at the onset of acute coryza, and the duration of the disease appears to be considerably reduced by the administration two or three times daily of one-half to one milligram. In chronic nasal catarrh, however, he could not find any improvement resulting from this treatment.—*Deutsche Med. Zeit.*

---

**Detection of Bacillus Tuberculosis.**

Dr. M. B. Cartzell adds an easy method to the many staining processes now in use. A small quantity of sputum is thinly spread on a glass slide; it dries in a minute or two, and then is passed slowly through an alcohol flame several times. A drop or two of Gradle's fuchsin solution (carbolic acid, mxv; distilled water, fl ℥ ss; dissolve and add saturated alcoholic solution of fuchsin, fl ʒ ss) is placed on the spectum for from three to five minutes. The slide is now washed with distilled water to remove excess of fuchsin, and the sputum is decolorized by sat. sol. of oxalic acid. It is again thoroughly washed in distilled water and allowed to dry; it is now ready to be mounted in glycerine or balsam. With a power of 500 diameters the bacilli appear as brilliant red rods, without staining the background. Nitric acid, employed in most other methods as a decolorizer, is apt to remove the color from the bacilli.—*Phil. Med. Times.*

---

**Diseases of the Kidneys—Anæsthetics in.**

Dr. Laurence Turnbull dwells upon the great importance of attention to the condition of the kidneys and examination of the urine when an anæsthetic is to be administered. Many deaths, unaccountable otherwise, are due to this cause. In diseases of the kidneys, the blood being loaded with urea, anæsthetics almost invariably produce coma and death. He enumerates a considerable number of deaths from ether and hydrobromic ether, but very few from chloroform. Norris has reported two cases of death supervening unexpectedly from sulphuric ether after operations for cataract. Both recovered consciousness but died comatose, one in a few hours, the other after eighteen days; no organic lesion was found *post-mortem* except Bright's disease. Cases have also been reported by Emmet, Hunt and Montgomery, verified by *post-mortem* examination. The kidneys are the active agents, in eliminating ether from the blood, and if they are unable to perform this office, and if the skin is cold, moist and inactive, death will supervene by accumulation of mucus in the lungs, or congestion of the brain, in true Bright's disease of the kidneys. —*Med. and Surg. Rep.*

---

**Eye Diseases and Affections of the Female Generative Organs.**

The frequent occurrence of diseases of the eye during the progress of various disorders of the female generative apparatus has been noted by Dr. Rempoldi (*Journal de Medicine de Paris* Among the menstrual disorders which may be accompanied by conjunctivitis, simple or phlyctenular keratitis and iritis, the author mentions especially amenorrhœa. But suppression of the menses from various causes may also be attended by affections of the choroid, by optic neuritis, retinitis, and glaucoma. In the course of inflammatory diseases of the sexual organs are frequently observed iritis and sclerotitis, with trigeminal neuralgia. During pregnancy and lactation, Dr. Rempoldi has observed conjunctivitis and pannus. Among the diseases appearing toward the cessation of lactation, are noted corneal ulcerations, retinal hyperæsthesia, disturbances of accommodation, photophobia, and retinitis. The author includes hysteria in the list of sexual disorders, and mentions asthenopia with retinal hyperæsthesia, and ptosis with retinal anæsthesia, as having been observed at different times in hysterical subjects. Finally, he notices the ocular disturbances dependent upon the albuminuria of pregnancy, and amblyopia consecutive to uterine hemorrhages.—*Med. Record.*

---

**Malignant Tumors Converted into Innocent Growths.**

Professor V. Nussbaum, in a clinical lecture recently delivered in Munich, expressed the belief that he had discovered a procedure for the positive cure of cancer by restraining the poliferation of the tissue elements of the disease. It appears to him that a total interruption of all peripheral sources of nutrition is the means best adapted to secure this result. He accomplishes this object by the use of the thermo-cautery, with which instrument a deep channel is made quite around the malignant growth, thus cutting off entirely the supply of blood and other nutritive fluids from the surrounding tissues. The small vessels which ascend into the tumor from the parts beneath are sufficient to preserve its vitality, so that gangrene does not occur. He thinks the thermo-cautery far preferable to the ligature, and that it possesses many advantages over the knife. He regards the hot iron and the various chemical caustics worthy of more extensive employment in the domain of more malignant growths than they have ever enjoyed. Prof. Nussbaum doubts not that thus circumscribing a cancerous growth, thus cutting off every channel of peripheral nutrition, has a brilliant future, especially in those desperate cases in which death is imminent from hemorrhage. In his experience this method of cutting off the peripheral blood supply has afforded such astonishing results that he recommends this procedure to the attention and practice of all those having occasion to treat a case adapted to its employment.—*Lancet.*

**Piles.**

The following formula is recommended in a recent issue of the *Gazette Medicale de Paris:*

℞

Iodoformi, ʒ j.

Balsam peru., ʒ ij.

Ol. theobromœ,

Ceræ alb., aa ʒ iss.

Magnes. calcin., ʒ j. M.

Divid. in suppos. No. xij.

One of these should be introduced after each evacuation.—*Med. and Surg. Reporter.*

**Epithelial Cancer.**

Dr. William A. Collins had a case of cancerous affection of the face in which the use of a knife or cautery was precluded. It occurred to him to try the effect of ergot to diminish the vascularity and capillary circulation, and thus limit the excessive cell-growth which forms the characteristic feature of these conditions.

*Fresh* ergot, *freshly* ground to an *impalpable* powder, was applied dry, by means of a soft hair pencil to the whole surface of the ulcer thrice daily. The ulcer was washed off carefully once daily. After each application the ulcer was covered with a light muslin rag, wet with the following lotion:

℞.

Acid carbolici, ʒ i.

Acid sulphurosi, ℥ j.

Glycerinæ, ʒ iv.

Aquæ, ℥ ijss.

M. S. Lotion.

Tonics were administered. In twelve weeks the whole ulcer had healed, and has remained so now for four years. In a case of mammary cancer, where an ulcer five inches in diameter and one inch deep occupied the site of the destroyed gland, and the axillary glands were involved, an equally complete though less rapid cure was obtained.—*Cinn. Lan. and Clinic.*

**Cure of Hemorrhoids by Carbolic Acid Injections.**

At a recent discussion of the New York Clinical Society on the treatment of hemorrhoids, Dr. C. B. Kelsey referred to his experience with injections of carbolic acid. He uses a solution of one part of pure carbolic acid in six and a half parts each of water and glycerine, and of this he injects about five drops into each hemorrhoidal tumor. For the operation no anæsthetic is required, and the subsequent pain is said to be very slight. In one case in which he used a solution of double strength, each pile sloughed; and in another case treated by one of his colleagues, Dr. Kelsey saw extensive ulceration caused, but this he attributes to want of skill in the manipulation. He has usually repeated the injection at the interval of about a week, so as to see the full effect of each injection before making another, and in this way the treatment may extend over months; but as it is painless and does not necessitate rest or even abandonment of ordinary work, this is not a serious objection to the plan in many cases. This mode of treatment, then, when carefully carried out, appears to be safe, efficient, nearly if not quite painless, and not requiring rest or special nursing. It may be useful, therefore, where for any reason the ligature or clamp operation cannot be performed, and especially where it is important not to lay the patient up during treatment.—*Lancet.*

[Equal parts of Calvert's solution of carbolic acid and olive oil has done excellently well in our hands, applied in the same manner. It is painless and thoroughly effective.—ED. BISTOURY.]

**Bromide of Nickel.**

Dr. DaCosta, in the Philadelphia *Medical News*, advocates the use of a new salt, bromide of nickel, in epilepsy.

He gives doses of five grains in form of pill or syrup, three times a day, gradually increasing the dose to ten grains, three times a day. A practical formula for the pills is as follows:

℞

Bromide of nickel, gr. lx.
Powd. althæa, gr. vi.
Extract of gentian, gr. vi.
Alcohol, q. s.

Mix and make twelve pills.

FOR SYRUP.

℞

Bromide of nickel, gr. 160.
Glycerine, ℥ ss.
Water to make, ℥ iv.

Dissolve the nickel in the water and add the glycerine.

Throw eight ounces of loaf sugar into a glass perculator and pour on the solution; pour back the syrup until the sugar is entirely dissolved, and make the measure eight fluid ounces with simple syrup.

This makes a handsome, green-colored, stable syrup, containing five grains to two teaspoonfuls.

Bromide or hydrobromate of nickel can be easily made by gradually adding to carbonate of nickel, mixed with four times its weight of water, enough hydrobromic acid until effervescence ceases, heating the mixture to boiling, and adding a small quantity of the carbonate in excess.

This solution of the bromide is filtered and evaporated on a water-bath to dryness, in a porcelain evaporating dish, stirring constantly with a glass rod. It should be kept in tightly corked bottles.

**Tongue in Health and Disease.**

Dr. A. W. Wallace condenses, in the September number of the *Midland Medical Miscellany*, Dr. Beale's remarks on the tongue, as contained in his admirable little work on slight ailments. A healthy tongue is best known by negative characters, rather than by what it is. In order, therefore, to define the healthy tongue the following conditions are to be excluded: First, the creamy white tongue, which denotes unremoved epithelium and metabolism of tissue in abeyance. Second, the furred tongue in which the papillæ are elongated, and to which the epithelium adheres in long threads; this tongue is characteristic of inflammation. Third, the pale, sodden, tooth-marked tongue, which is indicative of anæmia. Fourth, the red tongue (*a*) with enlarged papillæ, as seen in the "strawberry tongue" of scarlatina; (*b*) the smooth and glazed, as in the "irritable tongue," which corresponds to the irritated mucous membrane elsewhere, as in the lung from phthisis, or intestines from diarrhœa. Fifth, the dry, brown tongue. pathognomonic of the typhoid state, in which blood exudes and dries on its surface, the secretion of saliva being nil. Sixth, the aphthous tongue; which is often followed by "punched out" ulcers. This condition of the tongue is not particularly significant of any constitutional disturbance. It is more properly a local affection, and is to be treated with the chlorate of potash. And seventh, the red, fissured tongue, which is generally called syphilitic. Dr. Beale, however, says that this is not necessarily syphilitic, although he has found that the exhibition of the iodide of potassium, with or without small ($\frac{1}{32}$ of a grain) doses of biniodide of mercury is the most successful treatment.

**Carbolized Sawdust.**

Mr. H. P. Symonds, Surgeon to Radcliffe Infirmary, advocates the use of this material as a dressing for wounds. He says: One of the drawbacks of the usual antiseptic dressing is the rapidity with which the discharges come through on the first day or two after operation, often necessitating the re-dressing of the case within a few hours. To prevent this, and yet not to interfere with the aseptic condition of the wound, is a distinct advantage both to the patient and the surgeon. The material I have used recently in a considerable number of cases is coarse sawdust soaked in (1 in 10) solution of absolute phenol and spirit of wine, then allowed to dry slightly so that the spirit may evaporate, leaving the sawdust charged with carbolic acid. When used it is enclosed in a bag made of several layers of gauze, and applied outside the deep dressing, the usual external dressing being put over it. The sawdust thus takes the place of the padding of loose gauze which is generally used. Its absorbent power is very great, and it has the additional advantage of keeping up an equable pressure on the divided tissues. I find that fourteen ounces of sawdust will readily absorb about one pint of fluid.

He reports five cases, two of amputation, two of operation for tumor of the mammary glands, and one of compound dislocation of the elbow, in which it was used. In all these cases primary union took place without any formation of pus. In only one did the temperature reach 100°, and that on the day after operation, after which it became normal. I have not quoted these cases as being at all remarkable, but merely as common instances in antiseptic surgical practice in which the sawdust dressing was used. Surgeon-Major Porter, in *The Surgeon's Pocket-book*, states that he has used sawdust as a dressing in suppurating offensive wounds; but I am not aware that it has been tried, when prepared in the way that I have described, in antiseptic dressing. The three points in its favor are its powerful antiseptic qualities when saturated with carbolic acid, its great absorbent power, and its adaptability to any surface. I may add that the sawdust should be coarse, as I find that if it is very fine it passes through the gauze and irritates the skin.—*Lancet.*

**Needless, Useless Coughing.**

There is in the world, says Charles J. Hare, in the *Brit. Med. Jour.*, a great deal of what I am accustomed to call "needless, useless coughing." Where secretion takes place in the bronchial tubes, it must sooner or later be brought up; and for this purpose some "necessary" coughing must take place, or the patient will choke. But, both in organic diseases and in slight inflammatory or irritative affections of the air passages, there is often an immense amount of useless coughing—useless, that is, as regards bringing up any laryngeal or bronchial secretion, and far worse than useless, because it wears out the patient, prevents sleep, and, moreover, increases the condition which gives rise to it, inasmuch as it lets the affected parts have no rest or peace. Now, the effects of opium are both local and general; and if in mucilage of acacia, or tragacanth, or in glycerine, or with a thick solution of confection of dog-rose, or honey, you give frequently from the one-fortieth to the one-twentieth of a grain of morphia, you not only give a marvelous amount of peace and comfort to your patient, but, where it is remediable, you tend also to cure the disease. A favorite formula of mine, varied according to circumstances, is:

℞

Acetate of morphine, 1⅓ grs.
Nitric acid, dilute, 1½ drs.
Oxymel of squill, 6 drs.
Mucilage of acacia, 2½ ozs.
Glycerine, 2 drs.
Syrup of red poppy, 2 ozs.
Cinnamon of rose water sufficient to make the whole equal 6 ounces.

M. To take one or two teaspoonfuls five, six, or seven times in the twenty-four hours.

The coughing in pertussis may be similarly relieved.

**Pulmonary Congestion.**

Dr. Benjamin F. Westbrook, in a paper in the *Medical Record*, records a case of abstraction of blood from the right heart, in a man of about fifty years of age, with almost instant relief, but, unfortunately, as it was used as a *dernier resort*, the relief proved only temporary.

In using the puncture he gives explicit description of the anatomical situation of the heart and valves, and the place where the tapping should take place.

The *right auricle* is the most available point for tapping, inasmuch as its position is less variable than that of the ventricle, and its accessible portion more globular, with a greater antero-posterior diameter to allow of free penetration of an instrument without danger of its passing through into the posterior wall. As an additional advantage, he also gives the following propositions:

First—The right auricle projects to the right about equally in the third and fourth intercostal spaces.

Second—Its perpendicular depth varies greatly according to its distention and the condition of the left heart and lungs.

Third—The projection to the right is greatest, and the perpendicular depth least, when the right heart is distended, as in a death from coma and asphyxia.

Fourth—The internal mammary vein, which lies upon the sternal side of the artery, is very constant in its course, and situated, on an average, about one centimeter external to the right border of the sternum.

Fifth—The anterior border of the right lung almost always extends inward beyond the border of the sternum, reaching, or even passing the median line in many subjects.

He gives preference to tapping in the third interspace, for two reasons:

First—Because it is much wider than the fourth, and the needle passes with less difficulty.

Second—Because the line of the fourth would direct the needle more toward the auriculo-ventricular opening, where it might come in contact with the tricuspid valve.—*Med. and Surg. Rep.*

---

POISONOUS COLORS—A decree, prohibiting poisonous coloring of certain articles of food, came into operation in Germany, on April 1. Poisonous colors within the meaning of the act are any containing antimony, arsenic, barium (except sulphate of baryta), lead, chromium (except pure chromic oxide), cadmium, copper, mercury (excepting cinnabar), zinc, tin, gamboge, picric acid. The use of packings colored with any of the above is likewise prohibited, and, except in certain varnishes, these must not be used for coloring playthings. The use of colors prepared with arsenic for the manufacture of paper-hangings, as well as that of pigments containing copper prepared with arsenic, and of matters containing similar colors for the manufacture of materials of dress, is prohibited.

